# Reconstructing the feeding ecology of Cambrian sponge reefs: the case for active suspension feeding in Archaeocyatha

**DOI:** 10.1098/rsos.230766

**Published:** 2023-11-22

**Authors:** Brandt M. Gibson, Max Chipman, Paolo Attanasio, Zaid Qureshi, Simon A. F. Darroch, Imran A. Rahman, Marc Laflamme

**Affiliations:** ^1^ Department of Chemical and Physical Sciences, University of Toronto Mississauga, Mississauga, Ontario, Canada; ^2^ Department of Earth & Environmental Sciences, Vanderbilt University, Nashville, TN, USA; ^3^ Senckenberg Museum of Natural History, Frankfurt, Germany; ^4^ The Natural History Museum, London, UK; ^5^ Oxford University Museum of Natural History, University of Oxford, Oxford, UK

**Keywords:** Archaeocyatha, Cambrian, computational fluid dynamics, reefs, suspension feeding

## Abstract

Sponge-grade Archaeocyatha were early Cambrian biomineralizing metazoans that constructed reefs globally. Despite decades of research, many facets of archaeocyath palaeobiology remain unclear, making it difficult to reconstruct the palaeoecology of Cambrian reef ecosystems. Of specific interest is how these organisms fed; previous experimental studies have suggested that archaeocyaths functioned as passive suspension feeders relying on ambient currents to transport nutrient-rich water into their central cavities. Here, we test this hypothesis using computational fluid dynamics (CFD) simulations of digital models of select archaeocyath species. Our results demonstrate that, given a range of plausible current velocities, there was very little fluid circulation through the skeleton, suggesting obligate passive suspension feeding was unlikely. Comparing our simulation data with exhalent velocities collected from extant sponges, we infer an active suspension feeding lifestyle for archaeocyaths. The combination of active suspension feeding and biomineralization in Archaeocyatha may have facilitated the creation of modern metazoan reef ecosystems.

## 1. Background

Modern metazoan reef ecosystems play a vital role as biodiversity hotspots, housing rich marine flora and fauna communities. Following the dominance of microbial reefs in the Tonian and Cryogenian, the first metazoan reefs emerged during the Ediacaran–Cambrian transition [1]. In particular, the early Cambrian (Tommotian, approx. 529 Ma) was characterized by some of the first undisputed examples of calcifying metazoan reefs, with archaeocyaths, an extinct group of sponge-like organisms, serving as primary reef frame builders [2,3]. These Cambrian reefs provided new ecological niches for animals to seek protection, attach to hard substrates, and take advantage of large concentrations of biomass, thus playing a pivotal role in the early diversification of animal life (e.g. [4]). However, the palaeobiology of archaeocyaths, in particular their feeding mode, remains poorly constrained. Elucidating archaeocyath palaeobiology is therefore key to understanding the structure and function of early Cambrian ecosystems, rates and patterns of nutrient transport, the palaeoecology and geobiology of the earliest metazoan reefs, and the extent to which archaeocyath reefs may have helped structure the Cambrian radiation event.

**Figure 1.**
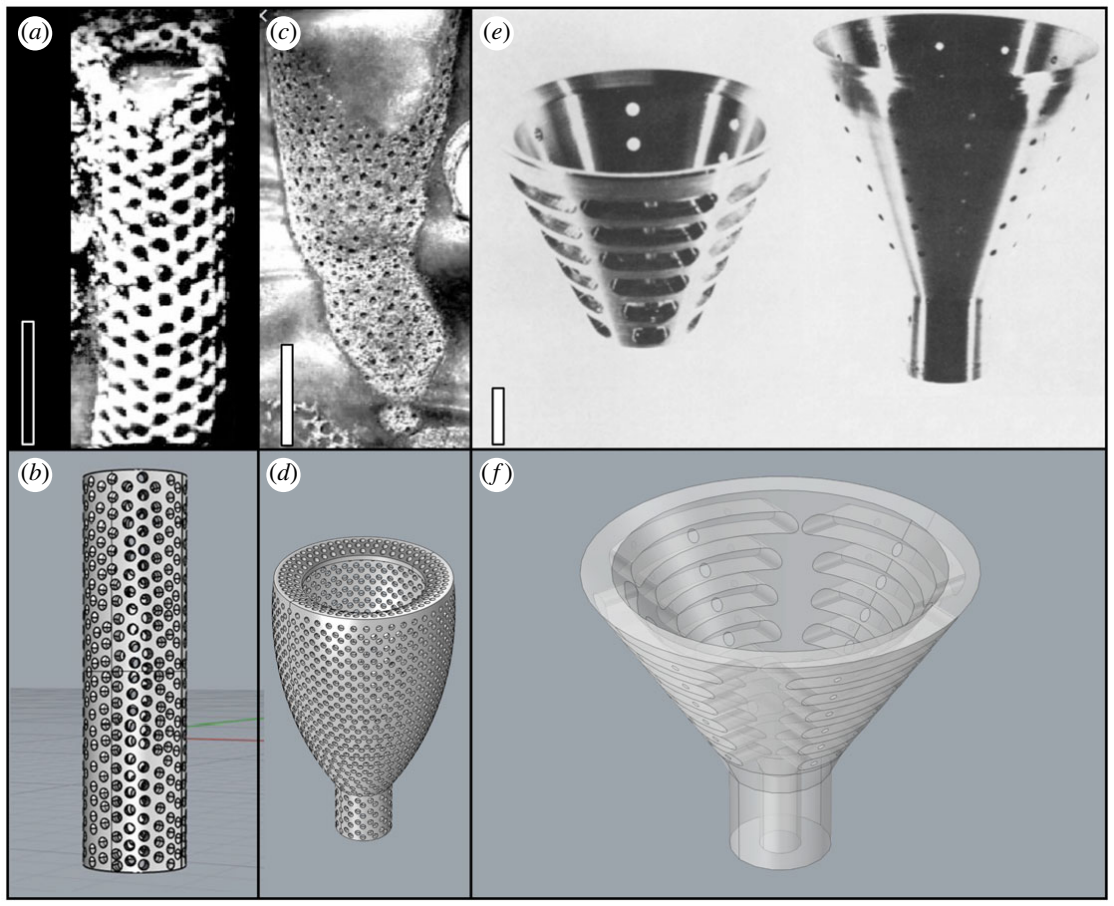
Three-dimensional digital models and images of the taxa they were based on. (*a,b*) *Favilynthus mellifer* [8]; (*c,d*) *Archaeolynthus porosus* [8]; (*e,f*) Balsam and Vogel models (BVM) and original aluminium BVM [12]. Scale bars are 5 mm for (*a,c*), and 10 mm for (*e*).

Archaeocyaths were globally distributed during the early Cambrian (approx. 530–509 Ma), forming metre-scale bioherms akin to modern patch reefs [3,4] to much larger constructions tens of metres in scale [5,6]. Most species are short (millimetre- to centimetre-scale), conical and characterized by porous skeletal walls [7–10]. Their calcite skeletons typically consist of inner and outer walls (‘cup in cup’ structure) connected by porous tabular (horizontal) and septal/taenial (vertical) partitions forming an intervening space termed the intervallum, which surrounds a larger central cavity that was probably open to the ambient ocean environment ([8,11]; figure 1). Their phylogenetic affinities have long proved contentious [13], with previous interpretations suggesting archaeocyaths were corals [14–16], algae [17,18], foraminiferans [19], sponges [20], or even belonging to their own kingdom [21]. However, current consensus, based on interpretations of their skeletal microstructure and the architecture of their porous (internal and external) skeleton, is that archaeocyaths represent calcifying sponges [8,13,22,23].

Given their porous morphology, archaeocyaths have been interpreted as macroscopic suspension feeders [8,9,13,23,24], wherein organic matter would have been extracted from water flowing through their pores and intervallum before exiting into the central cavity [13]. Modern sponges accomplish this using specialized choanocyte cells throughout their body, which beat flagella to force water through the canal structures within the walls and into chambers where feeding takes place through the mucus covered collar surrounding the flagella. Food particles stick to the mucus collar and are transported into the sponge tissue to be digested. The filtered water is then pushed into the central cavity and ejected out as an exhalent jet through the osculum. In concert with their pumping mechanisms, most sponges have morphologies that maximize the use of ambient currents to enhance circulation of water through their skeletons [12,25,26]. While most agree on a similar fluid movement pattern for Archaeocyatha [8,12,27,28], there is debate over whether this process was purely passive, similar to some crinoids, corals, and gorgonians, or if it required active filter feeding, similar to extant sponges, bivalves, and brachiopods [29]. Flume experiments performed by Balsam & Vogel [12] first suggested that archaeocyaths could have functioned as predominantly (or entirely) passive suspension feeders. However, subsequent studies have criticized their experimental design, pointing to their models' larger than natural sizes and inaccurate pore morphologies (e.g. [8,11,24]), both of which have a direct effect on current behaviour and particle transport. Here, we use computational fluid dynamics (CFD) of morphologically accurate models to revisit Balsam & Vogel's pioneering work [12] and assess whether archaeocyaths could have functioned effectively as passive suspension feeders. The results provide new insights into archaeocyath palaeobiology and carry implications for the function of Cambrian reef ecosystems.

## 2. Methods

### 2.1. Digital model construction

We used Autodesk Fusion 360 to construct digital models of three archaeocyaths (figure 1). All models were constructed as non-uniform rational basis spline geometries and are provided as the electronic supplementary material, S1a–c. Two single-walled archaeocyath taxa were chosen (cylindroconical-shaped *Favilynthus mellifer* and chalice-shaped *Archaeolynthus porosus*) based on their availability of detailed systematic descriptions, body and pore morphological descriptions, and published fossil photographs [8,30] (figure 1*a–e*; electronic supplementary material, table S1). We also replicated the double-walled idealized aluminium models used by Balsam & Vogel [12] (‘BVM’; figure 1*e,f*).

### 2.2. Computational fluid dynamics set-up

Archaeocyaths are reconstructed to have lived in a variety of conditions within open reefs, sheltered and/or lagoonal settings, and even more sheltered cavity dwellers [3,7,8,31,32]. Such disparate flow conditions suggest average ambient currents <10 cm s^−1^ for more sheltered conditions [33], and faster currents for reef crest communities [7] that would have been closer to 30–35 cm s^−1^ [33]. To account for this environmental variability, we simulated inlet flow velocities of *U* = 1, 5, 10, 15 and 30 cm s^−1^ for the three scaled models, informed by the characteristic flow velocities reported from modern coral reefs.

Three-dimensional, incompressible fluid flow simulations were performed using COMSOL Multiphysics. CFD is optimal for investigating small-scale flow patterns both within and surrounding the cavity and pores, and allows experimentation with multiple morphotypes and simulated flow velocities. We used hexahedral flow domains (electronic supplementary material, table S1) with the organism placed approximately one third of the length along the lower surface. The fluid properties of water (density (*ρ*) = 997 kg m^–3^, dynamic viscosity (*μ*) = 0.001 kg s^–1^ m^–1^) were assigned to the domain surrounding the model.

A stationary Reynolds averaged Naiver Stokes (RANS) shear stress transport (SST) turbulence model was used with a slip condition applied to the upper surface of the flow domain and sides parallel to flow. No-slip conditions were prescribed along the seafloor and organism model where velocity approaches 0 cm s^−1^. The flow domain was discretized into tetrahedral elements in the far field with hexahedral boundary layer elements in the vicinity of no-slip boundaries. A mesh sensitivity analysis was performed with an inlet velocity of *U* = 30 cm s^−1^. Our final meshes were selected based on a compromise between computational efficiency and accuracy (electronic supplementary material, figure S1). Because of the geometric and computational complexities of the *A. porosus* CFD simulations, the domain and model were bisected down the model's centre, permitting its symmetry to be used necessitating only half the domain to be solved.

To demonstrate comparability between methods, we replicated Balsam & Vogel's [12] physical experiments using their same Reynolds number corresponding to a water inlet velocity of 9 cm s^−1^ (that would have been a fourteen fold increase to 126 cm s^−1^ for air; electronic supplementary material, table S2; [12]). Because Balsam & Vogel's [12] experiments were inherently time dependent, we also completed 9 cm s^−1^ large eddy simulation CFD simulations for comparability using similar boundary conditions implemented in the RANS SST simulations [34]. Instead of a depth-averaged velocity profile prescribed to the inlet, we used the flow field from the RANS solution as our inlet condition. Flow fields were solved for 180 s to permit flow to fully develop before we exported flow fields every 0.01 s for 30 s.

## 3. Results

**Figure 2.**
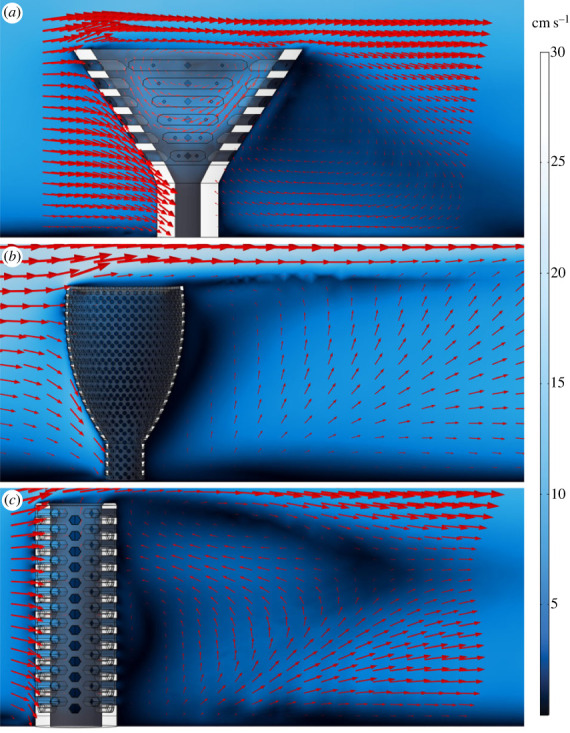
Fluid flow results for 9 cm s^−1^ inlet velocity for archaeocyath models. Red arrows show velocity magnitude and direction (*U*). All models are scaled to respective counterparts in figure 1. (*a*) Results for Balsam & Vogel [12] model. (*b*) Results for *Archaeolythus porosus* model. (*c*) Results for *Favilynthus mellifer* model.

**Figure 3.**
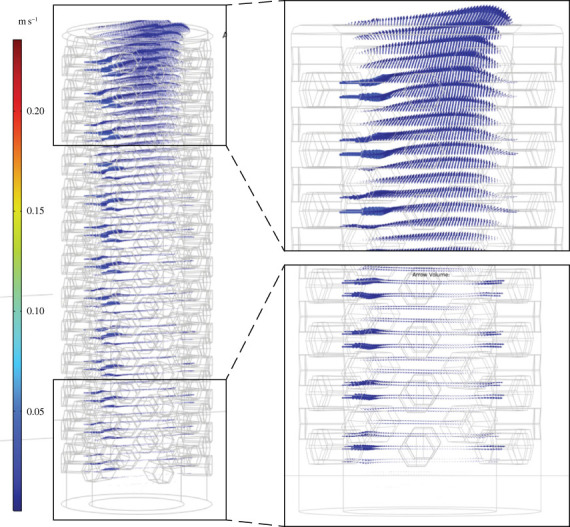
Flow velocity vectors (*U*) for *Favilynthus mellifer* CFD simulation with 9 cm s^−1^ inlet. Arrows scaled to 1.2x velocity magnitude of (*U*).

**Figure 4.**
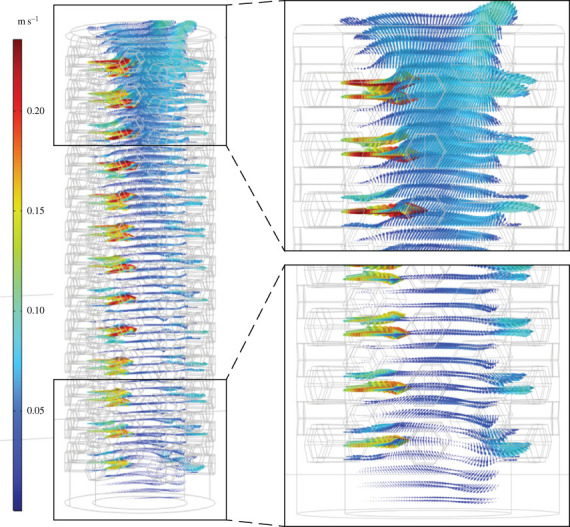
Flow velocity vectors (*U*) for *Favilynthus mellifer* CFD simulation with 30 cm s^−1^ inlet. Arrows scaled to 1.2x velocity magnitude of (*U*).

**Figure 5.**
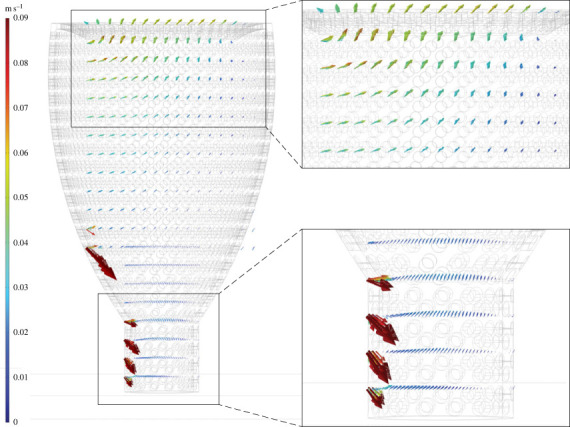
Flow velocities vectors (*U*) for *Archaeolynthus porosus* CFD simulation with 9 cm s^−1^ inlet. Arrows scaled to 3x velocity magnitude of (*U*).

**Figure 6.**
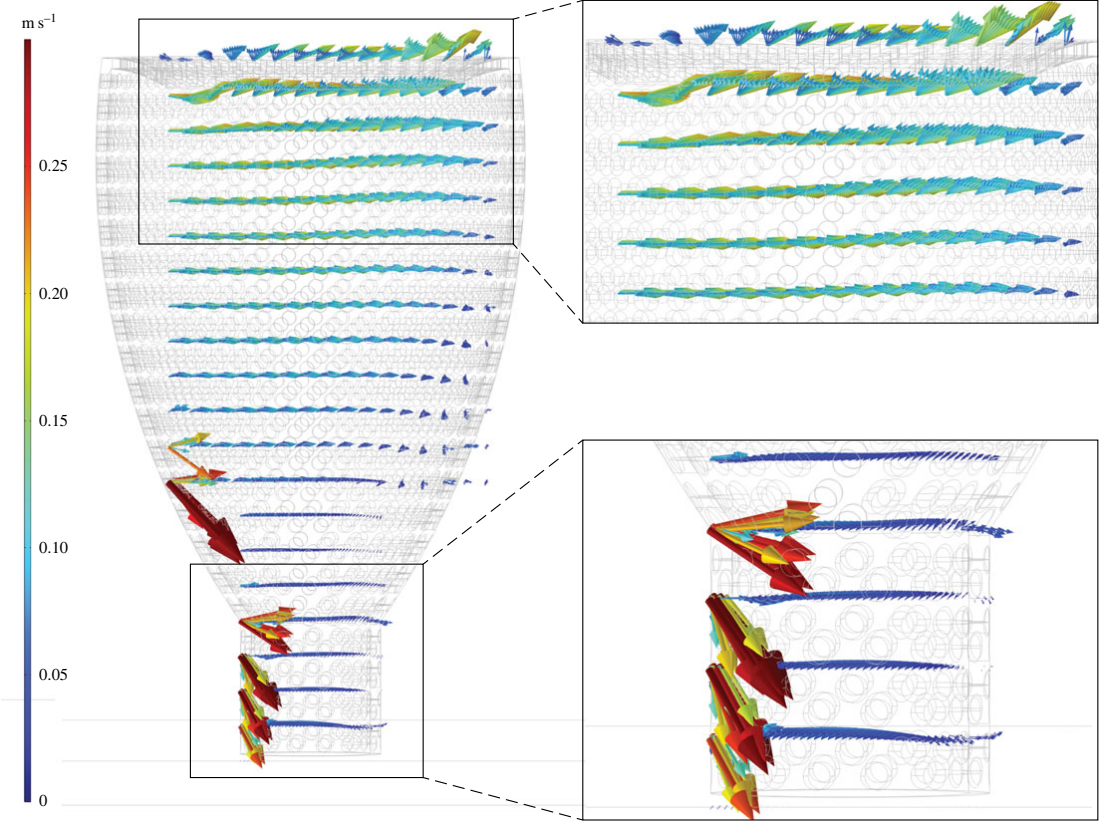
Flow velocities vectors (*U*) for *Archaeolynthus porosus* CFD simulation with 30 cm s^−1^ inlet. Arrows scaled to 3x velocity magnitude of (*U*).

**Figure 7.**
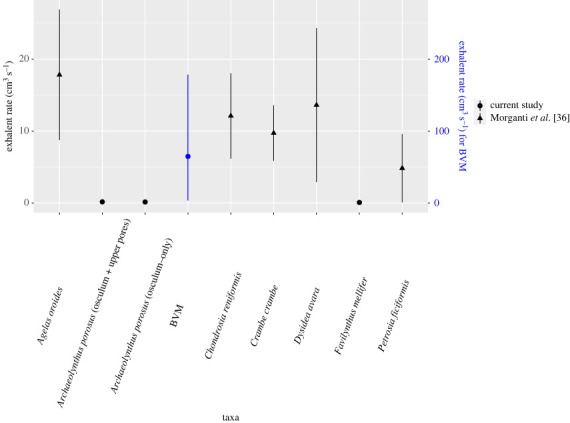
Typical ranges of osculum exit velocities (cm s^−1^) of extant sponges compared to the exit velocities from our archaeocyath models.

Some flow patterns were consistent across all simulations (figures 2–6; electronic supplementary material, figures S2–S4). A fully developed boundary layer with a characteristic viscous sublayer and Law of the Wall profile [35] formed upstream of the organism models. This pattern was disrupted by the models, and a wake formed directly downstream of the models. In all simulations, fluid flow was primarily directed around the models, with lower fluid volumes directed into the central cavities via the pores and from above the cavity. Flow velocity magnitudes (*U*) were slower in the cavity of the models than in the far field, and vortex shedding occurred off the upper surfaces of the models. Recirculation patterns within the wakes were evident downstream of the models, and the largest velocity magnitudes occurred within the upstream pores of the models. These patterns strengthened and weakened with increasing and decreasing inlet velocities, respectively. The lower velocity simulations (inlets < 15 cm s^−1^) developed viscous sublayers that were greater in vertical height (figure 2; electronic supplementary material, figures S2–S6). Because overall model morphology exerted such a strong control on flow pattern differences, we describe our results below organized into three morphotypes: cone-shaped BVMs; cylindroconical-shaped taxon (*F. mellifer)*; and chalice-shaped taxon (*A. porosus*). Lastly, exhalent velocity ranges for each species model are shown in figure 7.

### 3.1. Balsam and Vogel models

Velocity magnitudes within the central cavity were consistently lower than those exterior to the cup regardless of far field velocity (figure 2; electronic supplementary material, figure S2). Flow entered the cavity through the upstream pores, where maximum velocity magnitudes were observed at the intersection of the pores and cavity, which approached respective far field velocities. Some flow downstream of the individual was redirected upstream and into the central cavity through the downstream pores, though the pore velocity magnitudes were smaller than the upstream pore magnitudes (figure 2). Fluid within the cavity primarily exited via the osculum, though some flow ephemerally exited through the downstream pores (electronic supplementary material, figure S5). As observed in Balsam & Vogel's experiments [12], velocity magnitudes increased as flow moved from the outer pores to the inner pores into the central cavity, and pore velocity gradients were largest in the upstream pores (electronic supplementary material, figures S2 and S5). In these simulations flow can be observed exiting into the cavity via the upstream pores and being redirected vertically owing to greater pressure associated with the presence of the downstream cavity wall, which in turn leads to comparatively slower velocity magnitudes in the downstream pores (electronic supplementary material, figures S2 and S5). Osculum exit velocities were lower than the far field velocities in all simulations (figure 7).

### 3.2. Cylindroconical-shaped taxon *Favilynthus mellifer*

The cylindroconical *F. mellifer* velocity magnitudes for flow entering the cavities via the pores were slower than the far field velocity. Within the central cavity, most flow travelled horizontally and exited through the downstream pores, though some vertical movement occurred within the upper one-quarter to one-half into the central cavity and exited through the osculum depending on far field velocity (figures 3 and 4; electronic supplementary material, figure S3). Eddies were directed towards the base of the models near the downstream face and upwards out of the osculum near the upstream face (electronic supplementary material, figure S3). Internal flow was restricted to the upper one-third of the cavity, whereas the lower two-thirds was comparatively stagnant (figures 3 and 4; less than 1 cm s^−1^ with external currents of greater than 30 cm s^−1^). These cavity flow patterns were consistent for all far field velocities, though absolute cavity velocities scaled with far field velocity.

### 3.3. Chalice-shaped taxon *Archaeolynthus porosus*

Fluid flow for the chalice-shaped *A. porosus* was typically isolated to the upper third of the cavity with the remainder approximately stagnant. A larger zone of circulation formed within the upper one-third of the central cavity, with water then redirected towards the model base along the downstream face, until it was forced upwards towards the upstream face, finally exiting through the osculum. The downward flow of the circulation zones at the base of the model had the greatest absolute velocity within the skeleton (≥8 cm s^−1^ for the 30 cm s^−1^ far field velocity; figures 5 and 6). Only the slower (inlet 1–10 cm s^−1^) velocity simulations showed stagnation within the lower two-thirds of the central cavity.

## 4. Discussion

At one time, debate surrounded the ability of sponges to take advantage of passive flow. Vogel [25] proposed three mechanisms for induced current, two related to Bernoulli's equation and one related to viscous entrainment (see discussion in [26]), the latter being what has been attributed to archaeocyaths owing to their previously assessed conical shape [12]. As water flows over our models, we see much larger velocity flow interacting with the slower/nearly stagnant flow of water within the central cavity. Like the classic open cavity lid driven flow problem [37,38], the eddies within the model are created by higher velocity flow of water just above the osculum rather than the water entering through the pores. This suggests that much of the fluid flux into the cavity is a product of the flow above the model pulling water out of the cavity, thus requiring additional fluid to enter the cavity through the pores (Balsam & Vogel, [12]). While Vogel's assertion that greater ambient current velocities increase oscula exit velocities in sponges is intuitive, his experiments on both living and dead sponges have yet to be successfully replicated [26]. At first glance, our BVM simulations appear to match Vogel's assertions, with exhalent rates increasing with greater far field velocities, despite a lack of evidence for clearly defined effects of passive flow on extant, actively pumping, sponges [26]. This support dwindles when we apply the same principles to our fossil accurate models. Below we first discuss our CFD results in the context of Balsam and Vogel's original experiments, and then we broaden our discussion to incorporate fossil-accurate taxa.

Our BVM CFD results broadly replicate Balsam & Vogel's experimental results [12], which demonstrates the fidelity of the computational simulations. Overall, the CFD simulations reveal similar flow features, including many of the patterns described by Balsam & Vogel (figure 2; electronic supplementary material, figures S2 and S5; e.g. cavity circulation, downstream wake, faster pore velocities, etc.; [12]). Flow velocities within and exiting the central cavity in the BVM simulations were consistently higher than within the intervalum. This was probably caused by the friction from the added solid surfaces within the intervallum and the non-aligned inner and outer pores. These two features cause a large velocity reduction, and thus most of the inner cavity fluid circulation comes from water entering from above the organism. Moreover, the CFD results demonstrated consistent cavity recirculation and mixing, which was fuelled by faster velocity inflow through the pores and introduction from above the cavity (electronic supplementary material, figures S2 and S5). Ultimately, this would indicate limited stagnation within the central cavity of the BVM, which disappeared entirely at faster far field velocities. These results are in broad agreement with Balsam & Vogel's findings [12] and provide support for their interpretations of passive suspension feeding.

In terms of distinguishing between active and passive suspension feeding, we hinge interpretations on the circulation patterns observed within the archaeocyath skeleton. Fast fluid circulation would have facilitated feeding, while stagnation would indicate decreased access to nutrient-rich water [27]. Our experiments inherently assume a passive feeding model, and so by comparing exhalent rates (calculated as the exhalent velocity multiplied by the osculum surface area) in archaeocyaths with those known from extant sponges, we can establish if active pumping would have been required to produce similar exhalent rates. With, and probably because of, the greater body size and larger pore sizes of the BVM model when compared to a typical archaeocyath, the BVM CFD exhalent rates were much closer to expected sponge pumping rates [25,29,39–41]. Typical osculum exit velocities of extant sponges range from 5–27 cm^3^ s^−1^ in shallow reef environments with typical ambient flows of 0–36 cm s^−1^ (figure 7) [25,26,36,39,41,42]. The BVM developed maximum exhalent rates of 18 cm^3^ s^−1^ in a 30 cm s^−1^ flow environment, consistent with exhalent rates observed for extant sponges in similar conditions [25].

When assessing more accurate archaeocyath morphologies, modelled flow patterns share some similarities with key differences between the two morphologies (chalices and cylinders). Across the various inlet velocities, the *A. porosus* flow patterns more broadly matched the cavity recirculation patterns observed in the BVM results. All morphologies developed eddies within their respective cavities at the fastest inlet velocities. The BVM and *A. porosus* eddies were isolated to the upper cavity, while the *F. mellifer* patterns showed isolate circulation near its base. Also present in both morphologies, streamwise flow was observed in the lower *A. porosus* cavity region beneath the persistent eddy, and it was also observed in the central region of the *F. mellifer* cavity just above its respective eddy near the base. These flow disparities are unexpected for unified viscous entrainment feeding models. Across inlet velocities, very little vertical fluid movement out of the cavity into the overlying flow is present in the *A. porosus* models (figures 2, 5, 6; electronic supplementary material, figure S4), while much more appears present in the *F. mellifer* model results (figures 2–4; electronic supplementary material, figure S3)—offering the best evidence for vertical entrainment passive suspension feeding. As shown in figures 3–6, very little fluid actually moves through the single walled pores, with most fluid being directed around the organism model. Only in the faster velocities for the *F. mellifer* morphology do we observe consistent flow through the pores and then redirected towards the osculum and out into the ambient flow (figure 4).

When investigating the exhalent rates and flow velocities for the fossil-scaled archaeocyath models, we find exhalent rates consistently well below those of modern sponges. The cylindrical- and chalice-shaped models showed maximum exhalent rates of 0.2 cm^3^ s^−1^ and 0.8 cm^3^ s^−1^, respectively, while never surpassing the lowest end of typical exhalent velocities reported for sponges (for 30 cm s^−1^ far field flow conditions, figure 7) [25,26,36,39,41,42]. Furthermore, flow in the fossil-scaled models was isolated to the top third of the models, while the bottom third was nearly stagnant (<0.05 cm s^−1^) for most inlet velocities, suggesting that most of the water within the internal cavity was not effectively exiting the osculum, therefore preventing unfiltered water from passing through the porous walls/intervallum. At the slower far field velocities typical of lagoonal communities (less than 10 cm s^−1^; [33]), flow was approximately stagnant within the cavity with minimal movement through the pores. Given that several archaeocyath species are interpreted to have lived in calm settings with low ambient current velocities, such as in lagoons, reef cavities, and environments below fair-weather wave base [3,8], it is difficult to reconcile an obligate passive suspension feeding lifestyle with the anticipated metabolic requirements, unless large portions of the organisms became metabolically inactive through progressed ontogeny.

The combination of fossil scaled CFD results and exhalent rates of modern sponges suggest that if archaeocyaths were obligate passive suspension feeders, then they would require much slower metabolism or far greater concentrations of nutrients in the water column, the latter of which is unlikely given the prevalence of photoautotrophic reef dwellers in close association [2] and a reconstructed nitrogen cycle approximating modern oceans [43]. Alternatively, archaeocyaths could have employed some other form of nutrient gathering approach, such as photoautotrophic or chemoautotrophic symbionts that are known from extant sponges. However, geochemical studies suggest that photoautotrophic symbionts are unlikely as isotope ratios in the carbonate skeletons of archaeocyaths are at equilibrium with those inferred from sea water [8,44]. The most parsimonious explanation is that archaeocyaths behaved like their poriferan counterparts and made use of active suspension feeding methods.

We note that our models only replicate the archaeocyath skeletal tissues; in life, archaeocyaths would also have incorporated soft tissues enveloping the skeleton and probably within the walls and cavity. However, as our BVM two-walled comparisons have demonstrated, it is highly unlikely that this additional morphological complexity would have allowed for more efficient flow. We hypothesize that the soft tissue volumes filling additional space (and associated increase in texture) would probably have further slowed fluid flow through their skeleton and decreased accessible pore diameter even more, though further testing of this hypothesis is needed. Finally, owing to computational constraints on the modelling, most of our models represented single-walled archaeocyaths rather than the more common two-walled (cone-in-cone) morphology, which would have also further slowed the transfer of water from the external wall to the inner chamber, further reducing fluid circulation.

### 4.1. Implications

Reconstructing archaeocyaths as active suspension feeders has significant palaeobiological implications for understanding Cambrian reefs. Most extant sponges rely on actively pumping water through their skeletons, which has allowed them to expand to almost all marine environments and adapt to varying nutrient loads and sources [26,45]. An active feeding strategy would ensure archaeocyaths were not dependent on high energy environments (i.e. those modelled in our faster CFD analyses) in order to feed. Studies have shown (e.g. [3,46–48]) that archaeocyaths thrived in a variety of environments, from low energy deeper or sheltered reef mounds to high energy reef crests. The combination of a hard calcite skeletal framework in concert with metabolically pumped suspension feeding could have enabled the growth of larger archaeocyathid reef ecosystems. In addition to crafting new niches for themselves, archaeocyaths would have created a broad range of new niches for animals to diversify and fill that did not exist in the Ediacaran [49,50], potentially making these first large-scale metazoan reefs instrumental in the early diversification of Cambrian animals.

In comparison to modern reefs, archaeocyath reefs would have had a much greater proportion of active suspension feeders; although sponges, bivalves, and other active filterers are important components of Phanerozoic coral reefs [1]. With the presumed absence of photosynthetic framework reef builders in the early Cambrian ([51]; though see [52]), archaeocyath reefs were able to expand beyond oligotrophic waters within the photic zone, thus allowing these reefs to occupy deeper waters, much like modern glass sponge reefs [1]. Perhaps a better comparison for archaeocyath reefs would be stromatoporoid sponge reefs of the Palaeozoic, as they also would have been dependent on active suspension feeding and would have hosted many of the same reef dwelling organisms (echinoderms, brachiopods and large arthropods [2]).

## Data Availability

COMSOL set-up reports are reposited at Dryad Digital Repository: https://doi.org/10.5061/dryad.w3r2280xj [53]. Supplementary material is available online [54].
